# Decomposing door-to-needle time: insights into workflow delays and quality improvement in acute stroke care

**DOI:** 10.3389/fneur.2025.1651785

**Published:** 2025-08-01

**Authors:** Lianyan Wei, Hongfei Li, Zhuangzhuang Jiang, Dongjuan Xu, Xiaolan Wu

**Affiliations:** Department of Neurology, Affiliated Dongyang Hospital of Wenzhou Medical University, Dongyang, China

**Keywords:** acute ischemic stroke, door-to-needle time, intravenous thrombolysis, workflow optimization, physician experience

## Abstract

**Background:**

Timely administration of intravenous recombinant tissue plasminogen activator (rtPA) significantly improves outcomes in acute ischemic stroke (AIS). However, substantial variability in door-to-needle (DTN) time persists in real-world settings. This study aimed to deconstruct DTN time into distinct workflow intervals to identify key determinants of delay and inform targeted quality improvement.

**Methods:**

This retrospective study included 322 consecutive AIS patients treated with intravenous rtPA at Dongyang People’s Hospital between May 2023 and April 2025. DTN time was divided into four intervals: door-to-order time (ODT), order-to-imaging completion time (OCT), imaging completion-to-consent time (ICT), and consent-to-needle time (CNT). Real-time data were collected through beacon-based tracking and a time-tracking application. Linear regression, correlation, and subgroup analyses were used to explore factors associated with each interval and overall DTN time.

**Results:**

Among all intervals, ICT showed the strongest correlation with DTN time (*r* = 0.845, *p* < 0.01), followed by CNT and OCT. Imaging-guided thrombolysis significantly prolonged DTN time by 32.29 min (*p* < 0.0001), mainly through delays in ICT and OCT. Thrombolysis led by senior physicians was associated with a 7.61-min reduction in DTN time (*p* < 0.0001), driven by shorter ICT and CNT. MRI-negative strokes significantly prolonged DTN time by 6.28 min (*p* < 0.05), primarily due to a delay in ODT. Subgroup analysis revealed that junior physicians were more likely to cause delays during off-hours. Imaging-guided thrombolysis, such as CTP-guided and MRI-guided approaches, significantly prolonged DTN time due to extended OCT and ICT intervals (*p* < 0.001). Semiannual trends showed a gradual improvement in DTN performance until T3 (May–October 2024), followed by a plateau in T4 (November 2024–April 2025), possibly due to increased use of imaging-guided thrombolysis and more complex referrals.

**Conclusion:**

Physician seniority, thrombolysis strategy, and MRI-negative status significantly influence DTN time. Segmenting DTN time enables precise identification of key delays across different workflow stages and may enhance the efficiency of acute stroke care.

## 1 Introduction

Studies have shown that following the onset of cerebral infarction, approximately 1.9 million neurons are lost every minute (1). Compared with the normal rate of neuronal loss due to aging, this corresponds to an accelerated brain aging of about 3.6 years for every hour without treatment (2). Early administration of thrombolytic agents within the therapeutic window can substantially improve clinical outcomes (3, 4). To enhance acute ischemic stroke care, the American Heart Association/American Stroke Association (AHA/ASA) launched the Target: Stroke initiative in 2010, with the goal of reducing door-to-needle (DTN) time for intravenous recombinant tissue plasminogen activator (rtPA). Phase I of the program aimed to treat ≥50% of eligible patients within 60 min (5). In 2014, Phase II introduced more ambitious benchmarks: treating ≥75% of patients within 60 min and ≥50% within 45 min (6). Stroke quality improvement initiatives have also been implemented in several other countries. The Canadian Stroke Best Practices recommend an ideal DTN time of ≤30 min, with a maximum acceptable time of ≤60 min (7). In the United Kingdom and Australia, both the Sentinel Stroke National Audit Program and the Acute Stroke Clinical Care Standard set a DTN time target of ≤60 min. Germany’s Stroke Unit Certification Program requires that at least 80% of thrombolysis cases achieve a DTN time within 60 min (8). In China, stroke centers are categorized into two levels: the target DTN time is ≤60 min for primary stroke centers and ≤45 min for advanced stroke centers (9).

To achieve these goals, a range of streamlined strategies have been implemented across the acute stroke workflow. These include direct transfer of patients to the CT/MRI suite upon hospital arrival, pre-notification by emergency medical services (EMS) to alert hospital personnel prior to patient arrival, and rapid triage with immediate stroke team activation to expedite initial clinical assessment and imaging (6, 10). Concurrently, time-saving measures such as pre-mixing rtPA, initiating thrombolysis directly in the imaging suite, and simplifying or deferring the informed consent process have been adopted to further accelerate treatment initiation (11, 12). However, due to constraints in healthcare infrastructure and societal resources, it remains challenging to implement all recommended interventions consistently in routine clinical practice. In the United States, substantial regional variability in DTN time has been observed (13), largely reflecting differences in the adoption and execution of these strategies and stroke fast-track protocols. Consequently, identifying specific steps within the thrombolysis workflow that contribute most to treatment delays—and implementing targeted strategies to address them—is considered a highly effective approach to improving outcomes.

Numerous studies have focused on the acute stroke treatment process and have utilized real-time data collection through direct observations. These studies generally fall into two categories. One type evaluates the effectiveness of specific interventions in reducing DTN time by comparing DTN time before and after implementation (14–16); such studies may divide DTN time into components like door-to-imaging time and imaging-to-needle time. The other type aims to explore the influencing factors of DTN time as a whole, with only a few studies attempting a more detailed breakdown (17, 18). However, this approach may not fully capture the complexity of the workflow. Considering that the thrombolysis process requires coordination among multiple departments, a more detailed breakdown could offer a clearer understanding of the performance at each step. This study aimed to prospectively record time intervals throughout the thrombolysis workflow using a real-time data collection system and to further subdivide the DTN time into four distinct components, in order to identify key determinants of prolonged DTN time. By analyzing these intervals, we seek to pinpoint critical delays and provide evidence-based recommendations to further shorten DTN time and optimize the quality of acute stroke care.

## 2 Materials and methods

### 2.1 Patients

In this study, we retrospectively analyzed consecutive ischemic stroke patients who received intravenous rtPA thrombolysis at Dongyang People’s Hospital between May 1, 2023, and April 30, 2025. The study was approved by the Ethics Committee of Dongyang People’s Hospital and conducted in strict accordance with the principles of the Declaration of Helsinki. All personal information was anonymized during data collection and analysis to protect patient privacy. Inclusion criteria were as follows: (1) age ≥18 years; and (2) administration of intravenous rtPA thrombolysis in the emergency department. Exclusion criteria included: (1) in-hospital stroke cases receiving thrombolysis; (2) patients receiving tenecteplase (TNK) intravenous thrombolysis; and (3) incomplete or missing data.

### 2.2 Data collection

We collected a comprehensive set of baseline characteristics, including demographic information, vascular risk factors, and National Institute of Health stroke scale (NIHSS) scores based on medical records. Onset-to-door time, defined as the duration from the initial onset of ischemic stroke symptoms to hospital arrival, was obtained from the chief complaint section in the emergency department patient charts. Patients were categorized according to their mode of arrival—either self-presenting or transported by ambulance. Based on the time of arrival at the emergency department, patients were categorized as arriving during daytime hours (08:00–18:00) or outside of daytime hours. The physicians responsible for thrombolysis were categorized as senior if they were the leading physician in charge of daily clinical decision-making, and junior otherwise. The infarction site was classified as either anterior or posterior circulation based on magnetic resonance imaging (MRI) findings obtained after admission following thrombolysis. If lesions were present in both anterior and posterior circulations, classification was determined according to the patient’s primary symptoms. Patients with no new cerebral infarction lesions detected on post-admission MRI were classified as MRI-negative. Time-based thrombolysis referred to patients with a clearly defined symptom onset within 4.5 h, without the need for advanced imaging such as CT perfusion (CTP) or MRI. In contrast, imaging-guided thrombolysis was used for patients with wake-up stroke or those presenting 4.5 to 9 h after symptom onset, where CTP or MRI was necessary to guide treatment decisions. To accurately capture timestamps, beacon devices were installed at the emergency and radiology entrances to monitor patient flow throughout the stroke pathway. The admission time, order placement time, imaging scan time, and consent time were automatically recorded as electronic time stamps by the hospital information system, whereas the rtPA administration time was manually documented by medical staff. Combined with a time-tracking application and extraction of key time points from medical records, we precisely calculated the duration of each interval. The sum of all intervals constituted the DTN time. Data integration and analysis were conducted quarterly by teams from the emergency department, radiology department, and neurology department. Regular meetings were held to identify inefficiencies and implement targeted process improvements.

### 2.3 Stroke care pathway and time metrics

After hospital arrival, emergency physicians performed initial history-taking and physical examination. For patients suspected to be eligible for intravenous thrombolysis, the stroke fast-track pathway was activated immediately. The emergency physicians placed relevant orders and notified neurologists. Following blood sample collection, patients were escorted by stroke nurses to the radiology department for imaging. At our center, patients presenting within 4.5 h of stroke onset routinely undergo non-contrast CT (NCCT) and head and neck CT angiography (CTA), regardless of whether NCCT reveals ischemic or hemorrhagic stroke. For patients with wake-up stroke, or those presenting 4.5 to 9 h after symptom onset who might still be eligible for intravenous thrombolysis, NCCT and CTA are first performed. If NCCT excludes hemorrhage, additional imaging—such as CTP or MRI—is conducted to further assess eligibility for thrombolysis, based on mismatch findings between diffusion-weighted imaging (DWI) and fluid-attenuated inversion recovery (FLAIR) sequences on MRI, as described in the WAKE-UP study (19), or based on ischemic penumbra identified by CT perfusion (CTP) imaging, as referenced in the EXTEND trial (20). According to the TOAST classification (21), CTP is generally preferred when large artery atherosclerosis or cardiogenic embolism is suspected from clinical presentation and CTA findings. Conversely, MRI is usually favored if small vessel occlusion is suspected. Neurologists then arrived to reassess the patient, reviewed blood test and imaging results, and discussed the condition and treatment plan with the patient and family members. Intravenous rtPA was then administered by stroke nurses after informed consent was obtained. For patients with elevated blood pressure prior to thrombolysis, intravenous antihypertensive therapy was administered in accordance with Chinese guidelines for intravenous thrombolysis in ischemic stroke (22). Some patients underwent endovascular therapy following intravenous thrombolysis, depending on their clinical condition and family preference. In the stroke fast-track pathway, blood tests are expedited, and imaging data are generated promptly after scanning to enable quicker assessment. The DTN time was sequentially divided into four components: ODT: door-to-order placement time; OCT: order placement-to-completion of imaging time; ICT: completion of imaging-to-consent time; CNT: consent-to-needle time. The sum of all intervals constituted the DTN time.

### 2.4 Statistical analysis

All continuous variables were presented as medians with interquartile ranges (IQRs), as assessed by the one-sample Shapiro–Wilk test for normality. Categorical variables were expressed as counts and percentages. Baseline characteristics were stratified based on whether DTN time exceeded 45 min. The Mann–Whitney U test was used to compare continuous variables between groups, while the Pearson chi-square test was applied for categorical variables, as appropriate. To investigate factors influencing different intervals during thrombolysis, five separate linear regression models were constructed using the enter method. Although time variables were not normally distributed and typically require log transformation before regression analysis, similar results were obtained when using raw versus log-transformed data. Therefore, untransformed data were used for ease of interpretation and practical application. Spearman correlation analysis was performed to assess the association between each interval and the overall DTN time, aiming to identify the most impactful interval. Subgroup analyses further examined the impact of senior versus junior physicians on DTN time across different clinical scenarios. Kruskal–Wallis H tests were used to compare the components of DTN time across different time periods. The Bonferroni method was applied to adjust the *p*-values for pairwise *post hoc* comparisons. Statistical significance was defined as a two-sided *p*-value < 0.05. All statistical analyses were conducted using SPSS version 26.0, GraphPad Prism, and R software version 4.1.1.

## 3 Results

### 3.1 DTN time-based characteristics and correlations

**Figure 1:**
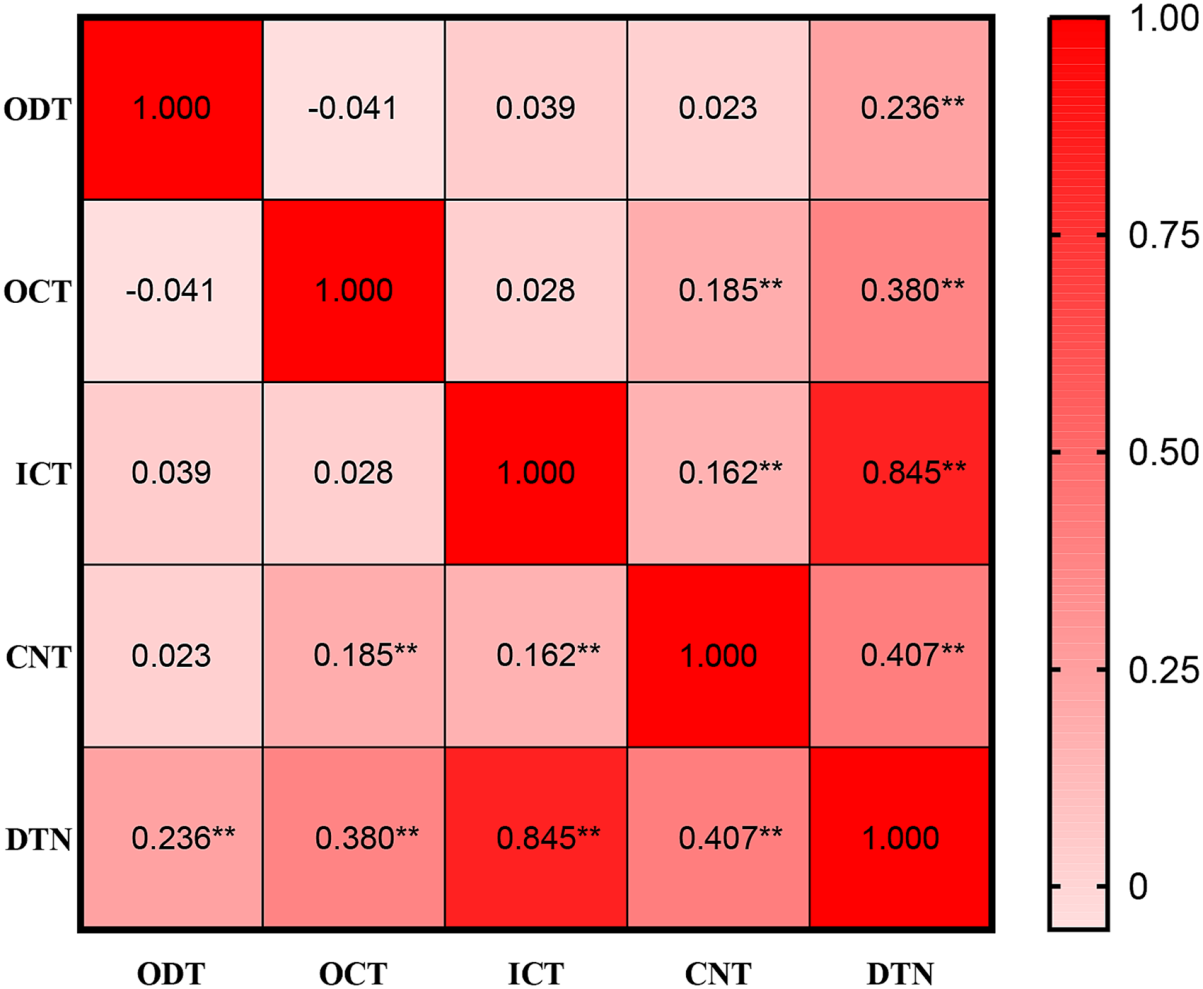
Spearman correlation matrix of time intervals. The strength of positive correlation with DTN time was ranked as follows: ICT (*r* = 0.870, *p* < 0.01), CNT (*r* = 0.402, *p* < 0.01), OCT (*r* = 0.377, *p* < 0.01), and ODT (*r* = 0.332, *p* < 0.01). In addition, CNT was positively correlated with both OCT (*r* = 0.112, *p* < 0.01) and ICT (*r* = 0.106, *p* < 0.01). DTN, door to needle; ODT, door to order time; OCT, order to imaging completion time; ICT, imaging completion to consent time; CNT, consent to needle time.

A total of 322 consecutive patients receiving intravenous rtPA thrombolysis were included. Table 1 presents baseline characteristics stratified by DTN time (≤45 min vs. > 45 min). No significant differences were observed in demographics, vascular risk factors, or most clinical variables between groups (all *p* > 0.05). However, thrombolysis led by junior physicians was more frequent in the DTN time >45 min group (38.00% vs. 25.23% *p* = 0.020), and imaging-guided thrombolysis (vs time-based) was significantly associated with delayed DTN time (17.00% vs. 0.90% *p* < 0.001). All internal time intervals showed significant positive correlations with DTN time (all *p* < 0.001). In Figure 1, among all intervals, DTN time showed the strongest correlation with ICT (*r* = 0.845, *p* < 0.01). In contrast, its correlation with ODT was the weakest (*r* = 0.236, *p* < 0.01). The correlations between DTN time and CNT (*r* = 0.407, *p* < 0.01) as well as OCT (*r* = 0.380, *p* < 0.01) were comparable. Interestingly, CNT exhibited weak correlations with both OCT (*r* = 0.185, *p* < 0.01) and ICT (*r* = 0.162, *p* < 0.01).

**Table 1:** Baseline characteristics of acute ischemic stroke patients treated with intravenous thrombolysis.

Variables	Total (*n* = 322)	DTN time≤45 min (*n* = 222)	DTN time >45 min (*n* = 100)	*p*
Demographic data				
Age (years), median (IQR)	70.00 (58.00, 78.75)	68.00 (57.25, 79.00)	72.00 (59.00, 78.00)	0.526
Sex, male, *n* (%)	206 (63.98)	143 (64.41)	63 (63.00)	0.807
Vascular risk factors, *n* (%)				
Hypertension	240 (74.53)	163 (73.42)	77 (77.00)	0.495
Diabetes mellitus	91 (28.26)	69 (31.08)	22 (22.00)	0.094
Hyperlipidemia	87 (27.02)	63 (28.38)	24 (24.00)	0.413
Ischemic heart disease	74 (22.98)	56 (25.23)	18 (18.00)	0.154
Atrial fibrillation	74 (22.98)	49 (22.07)	25 (25.00)	0.563
History of stroke	60 (18.63)	42 (18.92)	18 (18.00)	0.845
Mode of transport, *n* (%)				0.997
Private transport	219 (68.01)	151 (68.02)	68 (68.00)	
Ambulance	103 (31.99)	71 (31.98)	32 (32.00)	
Arrival time, *n* (%)				0.498
Other time	128 (39.75)	91 (40.99)	37 (37.00)	
Daytime (08:00–18:00)	194 (60.25)	131 (59.01)	63 (63.00)	
Physician seniority, *n* (%)				**0.020**
Junior physician	94 (29.19)	56 (25.23)	38 (38.00)	
Senior physician	228 (70.81)	166 (74.77)	62 (62.00)	
Infarct location, *n* (%)				0.689
Anterior circulation infarct	221 (68.63)	152 (68.47)	69 (69.00)	
Posterior circulation infarct	68 (21.12)	49 (22.07)	19 (19.00)	
Negative MRI	33 (10.25)	21 (9.46)	12 (12.00)	
Selection methods, *n* (%)				**<0.001**
Time-based thrombolysis	303 (94.10)	220 (99.10)	83 (83.00)	
Imaging-guided thrombolysis	19 (5.90)	2 (0.90)	17 (17.00)	
Reperfusion strategy, *n* (%)				0.861
IVT alone	272 (84.47)	187 (84.23)	85 (85.00)	
Bridging therapy (IVT + EVT)	50 (15.53)	35 (15.77)	15 (15.00)	
Antihypertensive pretreatment	59 (18.32)	40 (18.02)	19 (19.00)	0.833
Baseline NIHSS (scores), median (IQR)	5.00 (3.00, 10.00)	5.00 (3.00, 9.00)	5.00 (2.00, 12.00)	0.863
Time metrics (minutes), median (IQR)				
Onset-to-door	90.00 (60.00, 140.00)	90.00 (60.00, 130.00)	90.00 (60.00, 150.00)	0.424
Door to order	7.00 (5.00, 9.00)	7.00 (5.00, 9.00)	9.00 (6.00, 11.00)	**<0.001**
Order to imaging completion	9.00 (7.00, 12.00)	9.00 (6.00, 11.00)	10.00 (8.00, 14.25)	**<0.001**
Imaging completion to consent	17.00 (11.00, 26.00)	13.00 (9.00, 18.00)	29.00 (23.00, 38.00)	**<0.001**
Consent to needle	4.00 (2.00, 5.00)	3.00 (1.00, 5.00)	5.00 (3.00, 6.25)	**<0.001**

DTN, door to needle time; IQR, Interquartile range; MRI, magnetic resonance imaging; IVT, intravenous thrombolysis; EVT, endovascular thrombectomy; NIHSS, National Institutes of Health Stroke Scale. Values in bold are statistically significant at *p* < 0.05.

### 3.2 Determinants of DTN time in thrombolysis

Table 2 summarizes multivariable linear regression results. Imaging-guided thrombolysis prolonged DTN time by 32.29 min (95% CI: 25.48–39.10; *p* < 0.0001), driven by a 27.21-min delay in ICT (95% CI: 21.72–32.71; *p* < 0.0001) and 2.74-min delay in ICT (95% CI: 0.40–5.08; *p* < 0.05). Senior physicians reduced DTN time by 7.61 min (95% CI: −11.00 to −4.21; *p* < 0.0001), attributable to shorter ICT (*β* = −4.37 min; 95% CI: −7.11 to −1.63; *p* < 0.01) and CNT (*β* = −1.71 min; 95% CI: −2.75 to −0.67; *p* < 0.01). MRI-negative strokes extended DTN time by 6.28 min (95% CI: 1.05–11.51; *p* < 0.05), mainly through prolonged ODT (*β* = 1.82 min; 95% CI: 0.38–3.26; *p* < 0.05). Ambulance transport (*β* = −1.63 min; 95% CI: −2.58 to −0.67; *p* < 0.01) and Bridging therapy (*β* = −1.88 min; 95% CI: −3.26 to −0.51; *p* < 0.01) shortened ODT, whereas atrial fibrillation prolonged it (*β* = 1.32 min; 95% CI: 0.24–2.41; *p* < 0.05). Higher NIHSS scores (*β* = 0.13 min/point; 95% CI: 0.04–0.22; *p* < 0.01) and longer onset-to-door time (*β* = 0.02 min/min; 95% CI: 0.01–0.03; *p* < 0.05) independently prolonged OCT. Hypertension shortened CNT (*β* = −1.22 min; 95% CI: −2.33 to −0.12; *p* < 0.05), but antihypertensive pretreatment increased it (*β* = 2.21 min; 95% CI: 0.97–3.45; *p* < 0.001).

**Table 2:** Multivariable linear regression analyses for DTN time and time segments with clinical predictors.

Variables	Door to imaging-order	Imaging-order to imaging completion	Imaging completion to consent	Consent to needle	DTN time
Age	0.01 (−0.03, 0.04)	0.01 (−0.04, 0.05)	−0.02 (−0.11, 0.08)	0.02 (−0.02, 0.05)	0.01 (−0.11, 0.14)
Sex, male	−0.12 (−1.03, 0.79)	0.19 (−0.95, 1.32)	0.33 (−2.34, 3.01)	0.04 (−0.98, 1.06)	0.44 (−2.88, 3.76)
Hypertension	0.43 (−0.56, 1.42)	−0.07 (−1.31, 1.17)	0.54 (−2.38, 3.45)	**−1.22 (−2.33, −0.12)** *	−0.33 (−3.94, 3.29)
Diabetes mellitus	−0.85 (−1.93, 0.24)	0.68 (−0.68, 2.03)	−1.36 (−4.54, 1.83)	−1.08 (−2.29, 0.13)	−2.61 (−6.56, 1.34)
Hyperlipidemia	−0.05 (−1.18, 1.08)	−0.64 (−2.05, 0.76)	0.72 (−2.60, 4.03)	0.77 (−0.49, 2.02)	0.79 (−3.32, 4.90)
Ischemic heart disease	0.22 (−0.83, 1.27)	0.20 (−1.11, 1.52)	−2.80 (−5.90, 0.30)	0.32 (−0.86, 1.49)	−2.06 (−5.90, 1.78)
Atrial fibrillation	**1.32 (0.24, 2.41)** *	−0.32 (−1.68, 1.04)	0.59 (−2.60, 3.78)	−0.15 (−1.36, 1.06)	1.44 (−2.51, 5.40)
History of stroke	0.48 (−0.59, 1.56)	0.95 (−0.39, 2.30)	0.04 (−3.12, 3.20)	−0.50 (−1.70, 0.70)	0.98 (−2.94, 4.90)
Private transport	Reference				
Ambulance	**−1.63 (−2.58, −0.67)** **	0.37 (−0.82, 1.57)	1.26 (−1.56, 4.08)	0.37 (−0.70, 1.44)	0.38 (−3.12, 3.87)
Other time	Reference				
Daytime	0.44 (−0.43, 1.31)	−0.21 (−1.30, 0.87)	1.44 (−1.11, 4.00)	0.77 (−0.20, 1.74)	2.45 (−0.72, 5.61)
Junior physician	Reference				
Senior physician	−0.45 (−1.38, 0.48)	−1.07 (−2.23, 0.10)	**−4.37 (−7.11, −1.63)** **	**−1.71 (−2.75, −0.67)** **	**−7.61 (−11.00, −4.21)** ****
ACI	Reference				
PCI	−0.09 (−1.16, 0.98)	0.07 (−1.26, 1.41)	0.26 (−2.88, 3.40)	0.60 (−0.59, 1.79)	0.84 (−3.05, 4.73)
Negative MRI	**1.82 (0.38, 3.26)** *	−0.43 (−2.23, 1.36)	4.18 (−0.04, 8.40)	0.71 (−0.89, 2.32)	**6.28 (1.05, 11.51)** *
Time-based thrombolysis	Reference				
Imaging-selected thrombolysis	1.15 (−0.73, 3.02)	**2.74 (0.40, 5.08)** *	**27.21 (21.72, 32.71)** ****	1.19 (−0.89, 3.28)	**32.29 (25.48, 39.10)** ****
IVT alone	Reference				
Bridging therapy (IVT + EVT)	**−1.88 (−3.26, −0.51)** **	0.32 (−1.40, 2.03)	−1.05 (−5.08, 2.98)	1.34 (−0.19, 2.87)	−1.28 (−6.28, 3.72)
Antihypertensive pretreatment	−0.26 (−1.37, 0.85)	0.31 (−1.07, 1.70)	−1.12 (−4.38, 2.14)	**2.21 (0.97, 3.45)** ***	1.14 (−2.90, 5.19)
NIHSS scores	0.02 (−0.05, 0.10)	**0.13 (0.04, 0.22)** **	0.02 (−0.20, 0.24)	−0.01 (−0.09, 0.07)	0.16 (−0.11, 0.43)
Onset-to-Door time	0.02 (−0.01, 0.10)	**0.02 (0.01, 0.03)** *	0.01 (−0.01, 0.02)	−0.01 (−0.02, 0.01)	0.02 (−0.01, 0.04)

DTN, door to needle; ACI, Anterior circulation infarct; PCI, Posterior circulation infarct; MRI, magnetic resonance imaging; IVT, intravenous thrombolysis; EVT, endovascular thrombectomy; NIHSS, National Institutes of Health Stroke Scale.

Values represent linear regression coefficients (*β*) and 95% confidence intervals (95% CI). Statistical significance is denoted as: * *p* < 0.05, ** *p* < 0.01, *** *p* < 0.001, **** *p* < 0.0001.Values in bold are statistically significant at *p* < 0.05.

### 3.3 Physician seniority and DTN time delays

**Figure 2:**
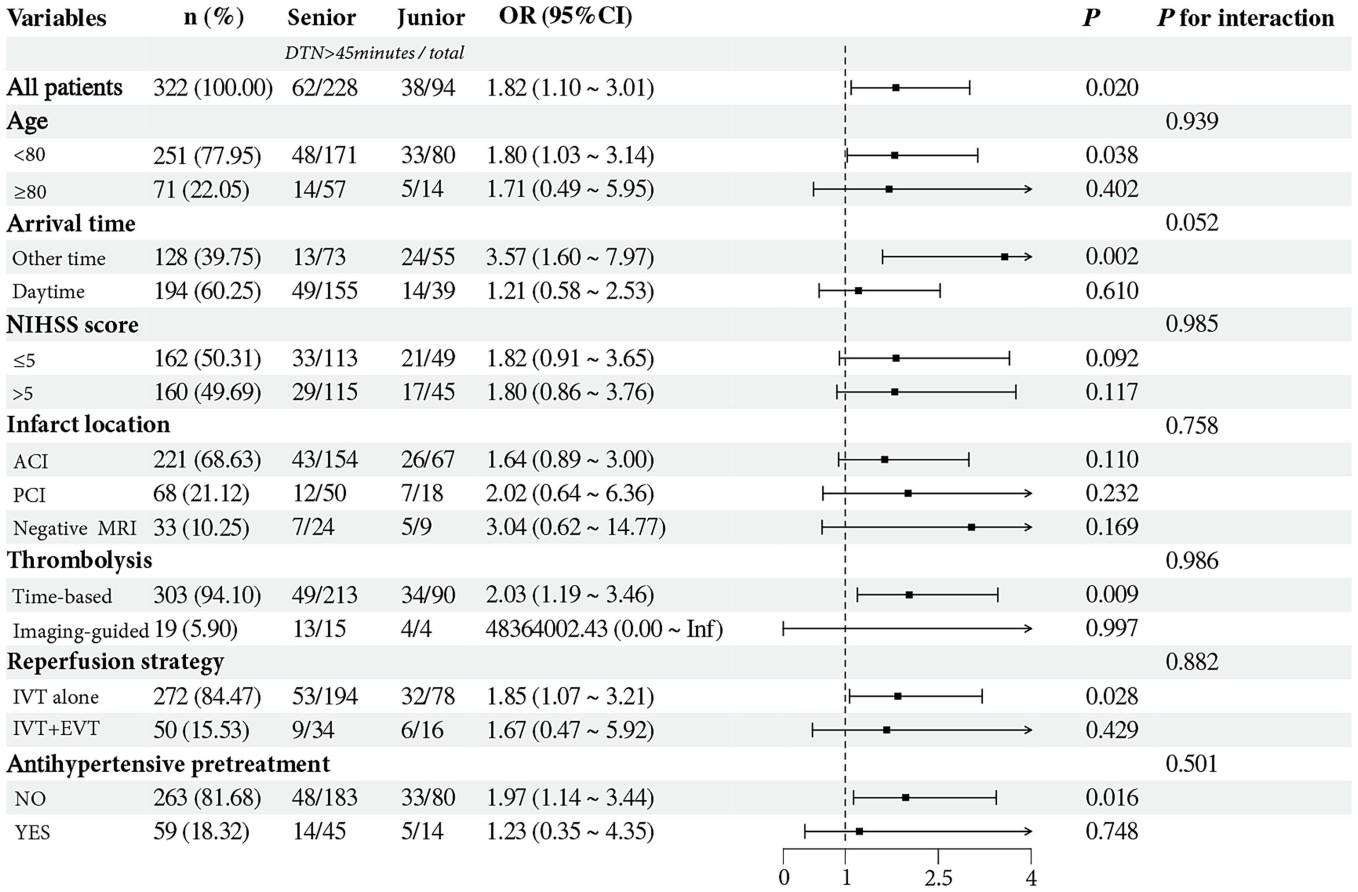
Subgroup analysis assessing the interaction effect of physician seniority on the likelihood of DTN time >45 min across various subgroups. The forest plot suggested that arrival time tended to modify the association between physician seniority and prolonged DTN time (*p* for interaction = 0.052). DTN, door to needle; NIHSS, National Institutes of Health Stroke Scale; Daytime, 08:00–18:00; AIC, anterior circulation infarct; PIC, posterior circulation infarct; MRI, magnetic resonance imaging; IVT, intravenous thrombolysis; EVT, endovascular thrombectomy.

Subgroup analysis was performed by stratifying patients according to age, time of arrival, NIHSS score, infarct location, thrombolysis selection method, reperfusion strategy, and antihypertensive pretreatment (Figure 2). Compared to senior physicians, junior physicians were more likely to be associated with treatment delays resulting in DTN time >45 min when treating patients who were younger (OR: 1.80, 95% CI: 1.03–3.14, *p* = 0.038), arrived outside daytime hours (OR: 3.57, 95% CI: 1.60–7.97, *p* = 0.002), received time-based thrombolysis (OR: 2.03, 95% CI: 1.19–3.46, *p* = 0.009), underwent intravenous thrombolysis alone (OR: 1.85, 95% CI: 1.07–3.21, *p* = 0.028), or had not received antihypertensive pretreatment (OR: 1.97, 95% CI: 1.14–3.44, *p* = 0.016). Notably, time of arrival tend to modify the association between physician seniority and achieving DTN time >45 min (*p* for interaction = 0.052).

### 3.4 Differences in thrombolysis time intervals across imaging strategies

Table 3 presents a comparison of thrombolysis-related time delays across different imaging strategies. Patients who underwent only NCCT had significantly shorter DTN time compared to those who received additional CTP (38.00 [30.50, 47.50] vs. 70.00 [58.75, 83.75], *p* < 0.001) or MRI (38.00 [30.50, 47.50] vs. 75.00 [58.50, 80.50], *p* < 0.001). This difference was mainly attributable to shorter OCT and ICT. Specifically, compared to the CTP group, patients in the NCCT group had a shorter OCT (9.00 [7.00, 12.00] vs. 13.50 [12.25, 19.25], *p* = 0.012) and significantly shorter ICT (17.00 [11.00, 24.00] vs. 38.50 [30.00, 54.00], *p* < 0.001). Similarly, compared to the MRI group, ICT was also markedly shorter in the NCCT group (17.00 [11.00, 24.00] vs. 50.00 [36.00, 53.50], *p* < 0.001). There were no statistically significant differences in any time metrics between the MRI group and the CTP group.

**Table 3:** Differences in DTN time and components across imaging strategies.

Time metrics (minutes)	CT (*N* = 303)	CTP (*N* = 8)	MRI (*N* = 11)	*p*
ODT, median (IQR)	7.00 (5.00, 9.00)	7.50 (3.75, 11.50)	9.00 (5.50, 10.50)	0.648
OCT, median (IQR)	9.00 (7.00, 12.00) b	13.50 (12.25, 19.25)	11.00 (7.50, 14.00)	**0.012**
ICT, median (IQR)	17.00 (11.00, 24.00) bc	38.50 (30.00, 54.00)	50.00 (36.00, 53.50)	**<0.001**
CNT, median (IQR)	4.00 (2.00, 5.00)	3.50 (2.75, 5.25)	3.00 (3.00, 5.50)	0.856
DTN time, median (IQR)	38.00 (30.50, 47.50) bc	70.00 (58.75, 83.75)	75.00 (58.50, 80.50)	**<0.001**

DTN, Door to Needle; ODT, Door to Order time; OCT, Order to Imaging completion time; ICT, Imaging completion to Consent time.

CNT, Consent to Needle time; IQR, Interquartile range.

b, Compared to CTP group; c, Compared to MRI group. Bonferroni-adjusted *p*-values for pairwise comparisons. Values in bold are statistically significant at *p* < 0.05.

### 3.5 Differences in thrombolysis time intervals across study years

Table 4 demonstrates temporal changes in DTN time and its components. The best DTN time were observed during T3 (May–October 2024), with improvement in both median value (36.0 min vs. 42.0 min; *p* = 0.040) and the proportion achieving DTN time ≤30 min (34.9% vs. 15.0%; *p* = 0.012) compared to the initial period (May–October 2023, T1). The CNT showed a decrease since T1 (May–October 2023) (*p* < 0.001). However, the OCT in T4 (November 2024–April 2025) showed an increase compared to the preceding period (May–October 2024, T3) (10.0 min vs. 8.00 min; *p* = 0.038).

**Table 4:** Differences in DTN time and components across study years.

Time metrics (minutes)	T1 (*N* = 80)	T2 (*N* = 90)	T3 (*N* = 83)	T4 (*N* = 69)	*p*
ODT, median (IQR)	7.00 (5.00, 9.00)	7.00 (5.00, 10.00)	7.00 (4.00, 9.00)	8.00 (5.50, 10.00)	0.280
OCT, median (IQR)	9.00 (7.00, 12.00)	9.00 (5.75, 12.25)	8.00 (6.00, 11.00) d	10.00 (8.00, 13.00)	**0.038**
ICT, median (IQR)	19.00 (13.00, 26.75)	16.00 (10.00, 25.25)	16.00 (10.00, 26.00)	18.00 (11.50, 26.50)	0.495
CNT, median (IQR)	5.00 (4.00, 6.00) bcd	4.00 (3.00, 5.00) **c**	2.00 (1.00, 4.00)	3.00 (1.00, 5.00)	**<0.001**
DTN time, median (IQR)	42.00 (34.00, 49.75) c	39.00 (30.00, 48.00)	36.00 (27.00, 45.00)	39.00 (33.00, 51.00)	**0.040**
DTN time, *n* (%)					
≤30 min	12 (15.0%) **c**	23 (25.6%)	29 (34.9%)	12 (17.4%)	**0.012**
>30 min	68 (85.0%) **c**	67 (74.4%)	54 (65.1%)	57 (82.6%)	
≤45 min	52 (65.0%)	62 (68.9%)	63 (75.9%)	45 (65.2%)	0.406
>45 min	28 (35.0%)	28 (31.1%)	20 (24.1%)	24 (34.8%)	
≤60 min	74 (92.5%)	83 (92.2%)	74 (89.2%)	59 (85.5%)	0.444
>60 min	6 (7.5%)	7 (7.8%)	9 (10.8%)	10 (14.5%)	

DTN, door to needle; ODT, door to order time; OCT, order to imaging completion time; ICT, imaging completion to consent time; CNT, consent to needle time; IQR, Interquartile range.

T1 (May–October 2023), T2 (November 2023–April 2024), T3 (May–October 2024), T4 (November 2024–April 2025). b, Compared to T2; c, Compared to T3; d, Compared to T4. Bonferroni-adjusted *p*-values for pairwise comparisons. Values in bold are statistically significant at *p* < 0.05.

## 4 Discussion

To our knowledge, this is the first to apply advanced time-tracking tools to investigate the determinants of DTN time by dividing it into four distinct intervals. These intervals correspond to key phases of the fast-track thrombolysis workflow: stroke recognition, in-hospital transport and radiological support, physician–patient communication, and nursing implementation. This approach enables the identification of specific delays within the pathway, thereby facilitating targeted quality improvement measures.

The American Heart Association/American Stroke Association recommends DTN time of no more than 45 min (6). Previous studies have demonstrated that the experience of thrombolysis physicians can significantly reduce DTN time (23, 24). Consistent with these findings, our study showed that DTN times ≤45 min were more frequently associated with thrombolysis led by senior physicians. On one hand, senior physicians exhibit superior expertise in accurately diagnosing ischemic stroke and identifying the indications and contraindications for thrombolysis. On the other hand, symptomatic intracranial hemorrhage following intravenous thrombolysis negatively impacts long-term outcomes (25). rtPA remains relatively expensive and is not fully covered by medical insurance in parts of China (26). In the context of increasingly complex physician–patient relationships in China, these factors make the thrombolysis consent process particularly complex and sensitive (27). Effective communication is therefore essential for gaining patient trust and securing timely consent. However, compared to senior physicians, junior physicians may have less experience in stroke management and weaker communication skills, both of which may contribute to treatment delays. Linear regression confirmed a significantly shorter ICT when thrombolysis was led by senior physicians (by 4.37 min), supporting this hypothesis. Notably, junior physicians also showed longer consent-to-needle times (by 1.71 min). This may be attributed to senior physicians’ greater proficiency in pre-thrombolysis blood pressure management and their perceived authority, which may enhance team coordination and improve execution efficiency among nursing staff.

Advanced imaging evaluation has been associated with delays in initiating endovascular reperfusion therapy in acute ischemic stroke (28). Similarly, our study observed that imaging-guided thrombolysis was more frequently accompanied by DTN time greater than 45 min. This method also emerged as the strongest independent contributor to DTN time delay, resulting in an average prolongation of 32.29 min. Linear regression indicated that the delay was primarily driven by extended OCT and ICT. Several factors contribute to these delays. First, MRI and CTP require patient transfer to specific imaging suites, which increases transport time. Second, the processing of MRI and CTP scans take longer than non-contrast CT, and MRI may not always be readily available. These factors hinder the timely completion of imaging. In addition, unlike the rapid acquisition of non-contrast CT, both MRI and CTP require more time for image acquisition and reconstruction. Furthermore, compared with the widely adopted time-based thrombolysis, imaging-selected thrombolysis is a relatively novel approach. Physicians may be more cautious when interpreting imaging results and discussing treatment options with patients, which substantially extends the interval between imaging completion and obtaining informed consent. Fortunately, studies have demonstrated that clarifying staff roles, streamlining transport and approval workflows, and initiating thrombolysis directly in the imaging suite can effectively reduce DTN time in the context of multimodal imaging (29, 30).

Previous studies have indicated that imaging-to-needle time, rather than door-to-imaging time, is the main source of variability in DTN time and more frequently contributes to delays in the timely administration of rtPA for acute ischemic stroke (18). In our study, imaging-to-needle time was further subdivided into ICT and CNT, with ICT identified as the primary contributor to overall DTN time. This finding may be explained by two main factors. First, ICT constitutes the largest proportion of the overall DTN time, so even small changes in this interval can substantially impact the total treatment time. Second, unlike the other intervals, which typically follow standardized workflows, ICT involves greater complexity. It is influenced by multiple factors, including radiological interpretation, patients’ comorbidities, family members’ understanding, physician decision-making, and communication efficiency. This multifactorial nature contributes to high variability in ICT and underscores its pivotal role in determining DTN time. In addition, we observed positive correlations between CNT and both ICT and OCT. The association between CNT and ICT may reflect differences in physician communication styles and the impact of physician authority on team coordination, as previously discussed. The correlation between CNT and OCT, on the other hand, may suggest limitations in resource allocation. Since both intervals rely heavily on the involvement of stroke nurses, ensuring adequate staffing and optimizing their deployment could further help reduce DTN time.

Linear regression analysis further revealed that MRI-negative ischemic strokes significantly prolonged DTN time, primarily by delaying the time from hospital arrival to imaging order placement. This delay may be attributed to two factors: First, patients with MRI-negative strokes often exhibit milder symptoms (31, 32), potentially reducing the urgency perceived by emergency physicians; Second, their atypical clinical presentations can complicate early recognition, necessitating more time for history taking, physical examination, and in some cases, additional investigations to rule out stroke mimics (33). In our study, 10 patients (3.1%) were considered to stroke mimics, including 4 with functional disorders, 2 with seizures, 2 with peripheral nerve palsy, 2 with metabolic disorders, and 1 with migraine. This incidence is consistent with findings from previous studies (34). Similarly, in patients with atrial fibrillation, ODT delays may result from the need for emergency physicians to evaluate anticoagulation status and perform electrocardiographic assessments. In contrast, patients receiving bridging therapy typically present with more severe and classic stroke symptoms compared to those treated with IVT alone (35, 36), facilitating quicker identification and a more streamlined in-hospital workflow. Consistent with previous studies, ambulance transport was associated with a shorter ODT, but not with a reduced overall door-to-needle time DTN time (37). This finding may be explained by the fact that, although prehospital personnel can facilitate early stroke recognition through preliminary history-taking and physical examination, their role has limited influence on the core in-hospital workflow components of acute stroke management.

Prolonged OCT was observed in patients with higher NIHSS scores and longer onset-to-door times, likely due to the increased complexity of clinical evaluation. Severe deficits may impair cooperation during transport and scanning (38), while delayed arrivals often necessitate extended imaging protocols to assess eligibility for reperfusion therapy, thereby prolonging the OCT interval. In our study, pre-thrombolysis antihypertensive treatment due to significantly elevated blood pressure was associated with a prolonged CNT, which is understandable. However, patients with a history of hypertension tended to have shorter CNT, a finding that appears somewhat counterintuitive. We speculate that this may be because such patients typically present with elevated baseline and initial blood pressure upon arrival, leading medical staff to anticipate the need for blood pressure management and prepare accordingly. In contrast, patients without a history of hypertension may experience stress-induced blood pressure spikes during rapid transport, imaging, or communication, which are often unexpected. As a result, the medical team may be less prepared, leading to delayed antihypertensive intervention and prolonged CNT.

Physician seniority, as a modifiable factor, significantly influences DTN time, prompting us to conduct a subgroup analysis. The most notable finding was that during off-hours, DTN time was significantly longer under the care of junior physicians compared to senior physicians. A study focused on delays in individual subtasks within the Acute Stroke Protocol and found that DTN time was significantly longer when patients were treated by non-stroke neurologists or during non-working hours, compared to treatment by stroke neurologists or during routine working hours (39). Differences in physicians’ competencies were likely exacerbated by the relative scarcity of medical resources and support systems during these periods, which further highlights the impact of junior physicians’ limited experience. The role of an authoritative senior physician becomes even more critical to ensure the smooth functioning of the stroke fast-track pathway during off-hours. Implementing targeted training for junior physicians and optimizing the distribution of thrombolysis shifts between junior and senior staff may reduce this disparity and enhance the timeliness of stroke treatment.

Quarterly data integration and analysis were jointly conducted by teams from the emergency, radiology, and neurology departments. Regular interdisciplinary meetings were held to identify inefficiencies and implement targeted process improvements. From T1 (May–October 2023) to T3 (May–October 2024), a continuous decline in DTN time was observed, accompanied by a steady increase in the proportions of patients achieving DTN time ≤45 min and ≤30 min. However, this trend plateaued in T4 (November 2024–April 2025). One possible explanation is the increased adoption of imaging-guided thrombolysis during this period, prompted by updates in clinical guidelines, which may have introduced additional workflow complexity and prolonged DTN time. Additionally, since T2 (November 2023–April 2024), the overall number of thrombolysis cases has gradually declined, likely due to the growing capacity of primary hospitals to administer intravenous thrombolysis (40). As a result, more straightforward stroke cases may have been treated locally, while the more complex cases were referred to our center, potentially contributing to longer DTN time.

This study offers several notable strengths. First, it introduces an innovative approach by decomposing DTN time into four distinct intervals, allowing for the precise identification of contributing factors at each stage. Temporal analysis of these intervals further supports dynamic quality improvement. In parallel, examining the interrelationships among different time intervals may aid in identifying systemic inefficiencies within the stroke fast-track pathway. Second, compared to previous research, the study incorporates a broader and more novel set of variables, including physician seniority, thrombolysis strategy, infarct location, and reperfusion method. Nevertheless, several limitations should be acknowledged. As a single-center study, it is subject to potential selection bias. Additionally, the relatively limited sample size and observation period call for larger, multicenter studies with extended follow-up to validate and generalize these findings.

## 5 Conclusion

Physician seniority, thrombolysis strategy, and MRI-negative status significantly influence DTN time. Segmenting DTN time enables precise identification of key delays across different workflow stages and may enhance the efficiency of acute stroke care.

## Data Availability

The raw data supporting the conclusions of this article will be made available by the authors, without undue reservation.
